# Structural Characterization of *Haemophilus influenzae* Enolase and Its Interaction with Human Plasminogen by In Silico and In Vitro Assays

**DOI:** 10.3390/pathogens10121614

**Published:** 2021-12-10

**Authors:** Yesenia Osorio-Aguilar, Maria Cristina Gonzalez-Vazquez, Diana Elizabeth Hernandez-Ceron, Patricia Lozano-Zarain, Ygnacio Martinez-Laguna, Cesar Raul Gonzalez-Bonilla, Rosa del Carmen Rocha-Gracia, Alejandro Carabarin-Lima

**Affiliations:** 1 Posgrado en Microbiología, Laboratorio de Microbiología Hospitalaria y de la Comunidad, Centro de Investigaciones en Ciencias Microbiológicas, Instituto de Ciencias, Benemérita Universidad Autónoma de Puebla, Puebla 72570, Mexico; yesenia.osorio.aguilar@gmail.com (Y.O.-A.); crispi333@yahoo.com.mx (M.C.G.-V.); plozano_zarain@hotmail.com (P.L.-Z.); ignacio.martinez@correo.buap.mx (Y.M.-L.); rochagra@yahoo.com (R.d.C.R.-G.); 2 Licenciatura en Biomedicina, Facultad de Medicina, Benemérita Universidad Autónoma de Puebla, Puebla 72420, Mexico; ar_dinamics@hotmail.com; 3 Director de Investigación Educativa, Instituto de Salud para el Bienestar, Acapulco 39355, Mexico; c.gonzalez.bonilla@gmail.com; 4 Licenciatura en Biotecnología, Instituto de Ciencias, Benemérita Universidad Autónoma de Puebla, Puebla 72570, Mexico

**Keywords:** *Haemophilus influenzae*, enolase, plasminogen-binding protein, interaction, virulence factor

## Abstract

*Haemophilus influenzae* is the causal agent of invasive pediatric diseases, such as meningitis, epiglottitis, pneumonia, septic arthritis, pericarditis, cellulitis, and bacteremia (serotype b). Non-typeable *H. influenzae* (NTHi) strains are associated with localized infections, such as otitis media, conjunctivitis, sinusitis, bronchitis, and pneumonia, and can cause invasive diseases, such as as meningitis and sepsis in immunocompromised hosts. Enolase is a multifunctional protein and can act as a receptor for plasminogen, promoting its activation to plasmin, which leads to the degradation of components of the extracellular matrix, favoring host tissue invasion. In this study, using molecular docking, three important residues involved in plasminogen interaction through the plasminogen-binding motif (_251_EFYNKENGMYE_262_) were identified in non-typeable *H. influenzae* enolase (NTHiENO). Interaction with the human plasminogen kringle domains is conformationally stable due to the formation of four hydrogen bonds corresponding to enoTYR_253_-plgGLU_1_ (K2), enoTYR_253_-plgGLY_310_ (K3), and enoLYS_255_-plgARG_471_/enoGLU_251_-plgLYS_468_ (K5). On the other hand, in vitro assays, such as ELISA and far-western blot, showed that NTHiENO is a plasminogen-binding protein. The inhibition of this interaction using polyclonal anti-NTHiENO antibodies was significant. With these results, we can propose that NTHiENO–plasminogen interaction could be one of the mechanisms used by *H. influenzae* to adhere to and invade host cells.

## 1. Introduction

*Haemophilus influenzae* is a Gram-negative bacterium that normally colonizes the respiratory tract in humans. *H. influenzae* is subdivided into seven groups, including six that express distinct serotypes of capsular polysaccharide (a–f) which are referred to as typeable, and uncapsulated bacteria which have been termed non-typeable *H. influenzae* (NTHi). These bacteria cause both invasive and non-invasive infections [1,2].

*H. influenzae* type b (Hib) causes many severe infections, including sepsis, epiglottitis, pneumonia, and meningitis [3]; in contrast, NTHi is a major cause of mucosal infections, such as otitis media, sinusitis, conjunctivitis, and exacerbations of chronic obstructive pulmonary disease [4].

The initial step in the pathogenesis of disease due to NTHi involves the establishment of bacteria on the respiratory mucosa, and several studies suggest that NTHi can cross between cells and invade the subepithelial space [5]. This process can be favored by the degradation of components of the extracellular matrix (ECM). 

On the other hand, enolase (2-phospho-D-glycerate hydrolase) is an enzyme that catalyzes the reversible interconversion of 2-phosphoglycerate (2-PGA) to phosphoenolpyruvate (PEP) [6]; however, enolase is considered as a moonlighting protein [7], and with this multifunctionality, it has been shown that it can be exported to the cell surface of a variety of prokaryotic and eukaryotic cells [8]. The precise mechanism by which enolase is exported to the cell surface is unknown at present; the enolase sequence does not present a signal peptide, therefore it is described as a protein with nonclassical export [9]. Some authors have speculated that a hydrophobic domain within enolase might serve as an internal signal sequence, while others suggest that posttranslational acetylation or phosphorylation may control membrane association [10]. A study carried out by Boël in 2004 reported that a small fraction of *Escherichia coli* enolase, as well as other enolases, are covalently modified by their substrate (2-PG), specifically, the amino acid Lys341, which is in the active site of enolase in *E. coli*. This modification inhibits the activity of the enzyme but is essential for its export to the extracellular medium [11]. Moreover, when enolase is exposed on the cell surface, it can act as a plasminogen (Plg) receptor; this interaction is important to mediate the activation of Plg into plasmin (Plm) [12], a serine protease that promotes the degradation of the extracellular matrix (ECM). Some pathogens have used this protease system, Plg/Plm, to infect and invade host cells [13].

Plasminogen contains five kringle domains (K1–K5) which mediate its binding to enolase with accessible carboxyl-terminal or internal lysine residues [14]. Moreover, enolase has an internal motif (_248_FYDKERKVY_256_) responsible for plasminogen binding, which has been characterized in enolases from *Streptococcus pneumonia* [15,16], as well as from other microorganisms, such as *Streptococcus iniae* [8], *Leishmania mexicana* [17], *Bartonella henselae* [6], *Plasmodium* [18], and *Theileria annulata* [19].

To date, there have been no reports on *H. influenzae* enolase–human plasminogen interactions. However, a putative internal binding site has been identified in the enolase sequence of NTHi (_252_FYNKENGMY_260_) [20].

The aim of this study was to investigate the role of non-typeable *H. influenzae* enolase (NTHiENO) as a plasminogen-binding protein by in silico and in vitro assays. Both studies show that NTHiENO can establish interactions with human plasminogens.

## 2. Results

### 2.1. Modeling *H. influenzae* Enolase Structure

**Figure 1:**
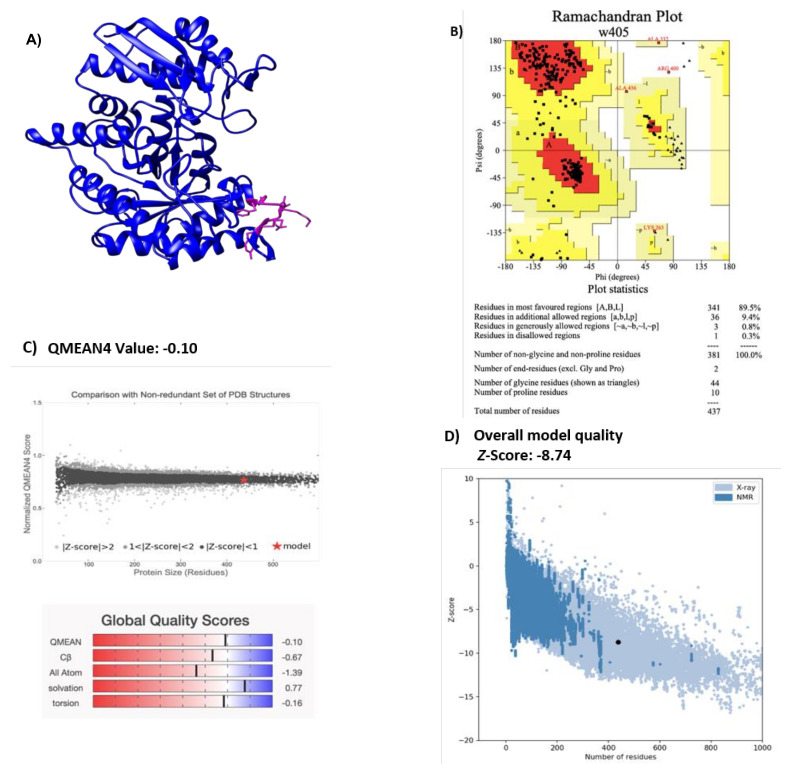
(**A**) Structural representation of NTHiENO model obtained by homology using Phyre2 and visualized by Chimera. Secondary structure, alpha helices, and beta strands are shown in blue; the putative plasminogen-binding site motif is shown in pink. (**B**) Ramachandran plot analysis of the theoretical model of NTHiENO. All residues except Gly and Pro are shown as square dots located in the most favored regions (89.5% in the red area), additional allowed regions (9.4% in the dark yellow area), generously allowed regions (0.8% in the light-yellow area), and disallowed regions (0.3% in the white area). (**C**) Normalized QMEAN score graphic showing the z-score value, and the position of the model of non-typeable *H. influenzae* enolase (red star) in the set of PDB structures used for evaluation. The Global Quality Scores are shown below: red (worse) and blue (better). (**D**) ProSA analysis (the black dot represents the model z-score for the protein of non-typeable *H. influenzae* enolase).

A structural description of the enolase of NTHi was obtained through homology modeling performed by Phyre2 [21], and the best-resolved structure obtained was visualized with the Chimera program [22] (Figure 1A). *E. coli* enolase was used as a template (Protein data bank: ID 2FYM) and has a resolution value of 1.60 Å [23]. This enolase sequence showed 85% of homology with NTHiENO. Due to the high identity of the template with NTHiENO, it provides a suitable template for modeling. The evaluation of the model by PROCHECK shows Ramachandran plot values of 99.7% (Figure 1B), indicating that the conformations of the amino acid residues are within the most favored or allowed regions. The Ramachandran diagram indicates that the stereochemical parameter Phi–Psi is ideal for the proposed model. The QMEAN value was -0.10, and the set of Z-values for different parameters, such as C beta interactions (-0.57), interactions between all atoms (-1.39), solvation (0.77), and torsion (-0.16), were very close to the value of 0, and this shows the fine quality of the model. The ProSA result obtained a model *z*-score of −8.74, which sits within the range of scores typically found for native proteins of a similar size—structures obtained by X-ray and NMR. (Figure 1D). The result set suggests that this model can be considered valid for the molecular docking assay.

### 2.2. Molecular Docking between NTHiENO and Plg

**Figure 2:**
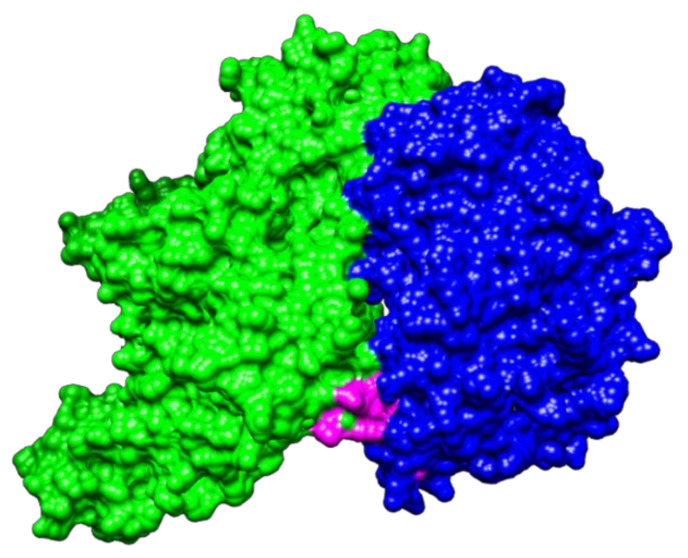
Visualization of protein–protein docking of NTHiENO and human Plg by PatchDock. Binding proteins are shown as surface representations. NTHiENO is shown in blue, the internal putative plasminogen-binding motif is shown in pink, and human Plg (4A5T) is shown in green.

The result obtained with PatchDock for protein–protein docking indicated that NTHiENO and Plg (4A5T) exhibited surface complementarity and that the putative plasminogen-binding motif is present in the interface area, suggesting that it could participate in the interaction (Figure 2). The ACE value of the model was -101.53 binding affinity (kcal/mol) of -21.3 and Kd (M) at 37.0 ℃ of 1.0 × 10^-15^.

### 2.3. Identification of Putative Plasminogen-Binding Domain in NTHiENO

**Figure 3:**
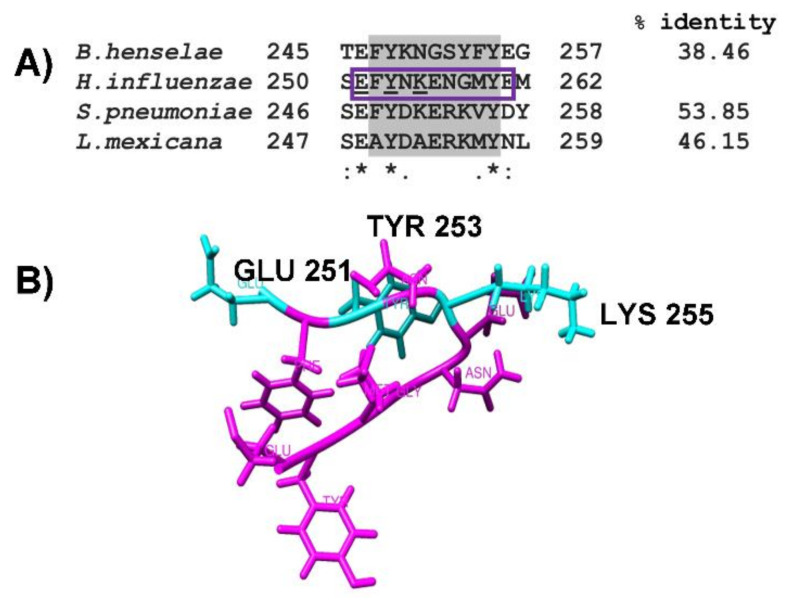
The region corresponding to the plasminogen-binding motif in different enolases. (**A**) Alignment of the internal plasminogen-binding motif described in *S. pneumonia*, *L. mexicana*, *B. henselae*, and the putative plasminogen-binding motif in non-typeable *H. influenzae* enolase, using CLUSTAL O (1.2.4) (highlighted in gray). The alignment showed 53.85, 46.15, and 38.46% of identity, respectively. The conserved amino acids in all sequences are labeled with asterisks; the conservative and semi-conservative substitutions are labeled with two points or one, respectively; pbmHiENO (purple box) and the proposed Plg binding residues are underlined. (**B**) shows the peptide of 11 amino acids (_1_EFYNKENGMYE_11_) (pbmHiENO) obtained with Gauss View 5.0 software, visualized by Chimera. Plg-binding residues are highlighted in cyan (GLU_251_, TYR_253_, and LYS_255_).

The alignment by CLUSTAL O, realized with enolases sequences, has characterized the plasminogen-binding motif and the putative motif present in NTHiENO corresponding to (_252_FYNKENGMY_260_), showing a high percentage of homology with the plasminogen-binding motif of *S. pneumoniae* (53.85%), followed by *L. mexicana* (46.15%) and a lower percentage of homology with *B. henselae* (38.36%). Although the plasminogen-binding motif presents some differences in the amino acid sequences, the main residues responsible for the interaction with enolase contain both positive and negative charges (Figure 3A). Only the region corresponding to the plasminogen-binding motif is shown in all aligned enolases. 

### 2.4. Obtention of pbmHiENO Peptide 

The plasminogen-binding site in enolases studied in the majority of pathogenic organisms is the C-terminal lysine [17]. It was observed that typeable and non-typeable *H. influenzae* enolase lacks C-terminal lysine [20]; however, it has the putative motif for plasminogen binding. Furthermore, this motif is exposed, so it could act as a plasminogen-binding site, as observed in the interface region of the protein–protein interaction carried out by PatchDock. For this reason, and to make the analysis more sensitive and specific, we performed an analysis by molecular docking (protein–ligand) to see if this motif (_252_FYNKENGMY_260_) has a role in the interaction with human plasminogen and to identify the important residues for this interaction. 

Thus, the secondary structure of NTHiENO was worked using Gauss View 5.0 software, and an 11 amino acid peptide was obtained: _1_EFYNKENGMYE_11_ (pbmHiENO). Equivalent to position 251–261 in the *H. influenzae* enolase sequence, this peptide contains an amino acid at both the carboxyl and amino-terminal ends to maintain the wild three-dimensional structure (Figure 3B). It was used as a ligand to perform blind docking assays.

### 2.5. pbmHiENO–Plasminogen Interaction

Plasminogen-binding sites with pbmHiENO were evaluated by blind docking, for which the crystal structure of human plasminogen that has already been determined and is available in PDB (4A5T) was used. The searches for conserved kringle domains in human plasminogen provided the following data: K1 (82–164), K2 (166–243), K3 (256–333), K4 (356–437), and K5 (460–541). These regions were used as a target for pbmHiENO by means of blind docking using the AutoDock Vina program.

**Figure 4:**
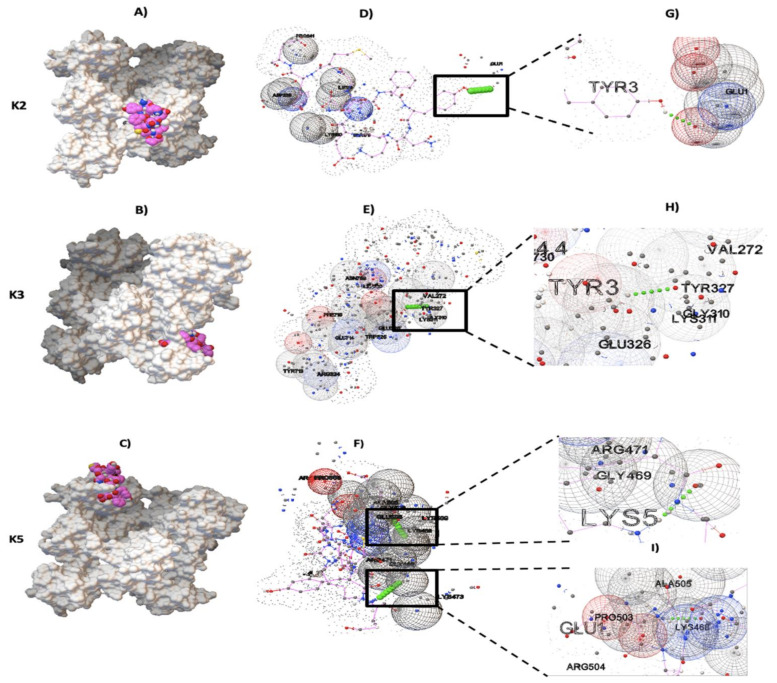
Plasminogen-binding sites with pbmHiENO using AutoDock Vina. (**A**–**C**) The protein–ligand interaction is shown in a surface representation, with pbmHiENO (shown in pink; the blue, red and yellow spheres corresponding to N, O and S atoms, respectively).) bound to kringle domains K2, K3, and K5 of plasminogen (4A5T) (shown in grey). (**D**–**F**) The interaction of pbmHiENO (dotted chain) with the kringle domains of Plg is shown by the formation of four hydrogen bonds (small green spheres in a square), along with other plasminogen residues involved in the interaction (grid spheres). (**G**–**I**) The important residues for the interaction correspond to K2: enoTYR253-plgGLU1, K3: enoTYR253-plgGLY310, and K5: enoLYS255-plgARG471/enoGLU251-plgLYS468. (These residues are equivalents to TYR3, LYS5, and GLU1 in pbmHiENO.)

Results for protein–ligand docking showed that pbmHiENO and human plasminogen had surface complementarity in the domains K2, K3, and K5 (Figure 4A–C). Multiple models were obtained; however, only the models that showed the best poses with the best binding affinity (kcal/mol) were chosen, as were the models with interactions characterized by the formation of hydrogen bonds.

According to molecular docking results, pbmHiENO showed greater affinity to K2, K3, and K5 domains, having a binding affinity kcal/mol of -3.9, -4.4 and -4.8, respectively. The pbmHiENO–Plg interaction presented conformational stability generated by hydrogen bonds and other intermolecular forces exerted by multiple residues of plasminogen (Figure 4D–F). 

In these analyses three important residues in NTHiENO were identified, essential for interaction with Plg through the formation of four hydrogen bonds corresponding to enoTYR_253_-plgGLU_1_ (K2), enoTYR_253_-plgGLY_310_ (K3), and enoLYS_255_-plgARG_471_/enoGLU_251_-plgLYS_468_ (K5), equivalents to TYR_3_, LYS_5_, and GLU_1_ in the pbmHiENO (Figure 4G–I). The summary of the data is shown in Table 1, and the rest of the amino acids of plasminogen that participate in the interaction are shown in Table 2. The molecular docking in the case of the interaction with the K5 domain was carried out before and after adding the caps, where we observed that the GLU1 and LYS5 residues are maintained in the formation of hydrogen bonds.

### 2.6. Docking Analysis in Positive Controls

In the Docking experiments with positive controls, the performance of the assays was measured by calculating the root mean square deviation (RMSD) between the coordinates of the atoms of the crystallized ligand structure and the docked pose using SPDV software. AutoDock Vina docked the complexes successfully. The docked conformation with values of the best-scored poses showed an RMSD to 89M/docked pose (0.61 Å), 0E7/docked pose (0.65 Å), and IMA/docked pose (0.68 Å). These low values of RMSD <2.0 Å, as well as the binding affinity presented by the positive controls (Table 3), provide information on the reliability of the software in the prediction of interactions observed for pbmHiENO and human plasminogen.

### 2.7. Experimental Detection of Non-Typeable *H. influenzae* Enolase–Plasminogen Interaction 

**Figure 5:**
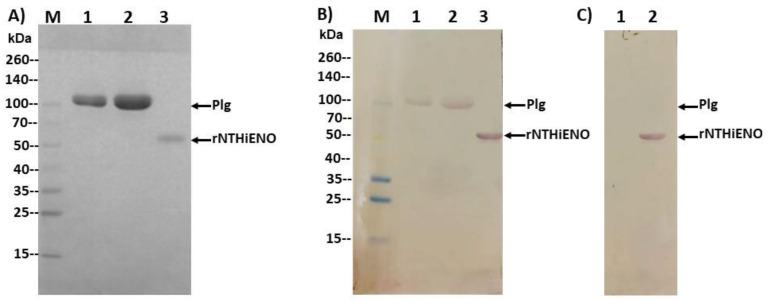
Identification of the binding of rNTHiENO to plasminogen by means of far western blotting. (**A**) Protein migration in SDS-PAGE: 10%. M: weight marker; Lane 1: Plg (5 μg); Lane 2: Plg (10 μg); Lane 3: rNTHiENO (3 μg). (**B**) Immunodetection of rNTHiENO–Plg interaction using polyclonal anti-rNTHiENO antibodies as the first antibody type, followed by incubation with IgG anti-mouse accoupled to alkaline phosphatase as the second antibody. After migration and electrotransference of proteins, and prior to the use of the antibodies, the blot was incubated with rNTHiENO. M: prestained protein marker; Lanes 1,2: rNTHiENO–Plg interaction; Lane 3: rNTHiENO. (**C**) A procedure like (**B**), but using only incubation with BSA as a negative control. Lane 1: Plg (5 μg), no signal was observed; Lane 2: rNTHiENO. The band corresponding to rNTHiENO (52.0 kDa) is indicated.

Once the interaction in silico and the probable residues involved in the interaction had been identified, the next objective was to demonstrate the protein–protein interaction in vitro. Far-western blot analysis was used to study the binding ability of NTHiENO to human plasminogen. Commercial human Plg or recombinant enolase of non-typeable *H. influenzae* (rNTHiENO) were migrated in 10% SDS-PAGE (Figure 5A) and electrotransferred to a nitrocellulose membrane. The membrane was blocked and subsequently incubated with rNTHiENO to continue with traditional western blotting assays. The rNTHiENO–Plg interaction was identified with mouse polyclonal antibodies. Anti-rNTHiENO was a signal corresponding to the plasminogen weight observed, as well as a signal corresponding to rNTHiENO weight as a positive control (Figure 5B). No positive signal was detected when the membrane was incubated with BSA as a negative control (Figure 5C).

**Figure 6:**
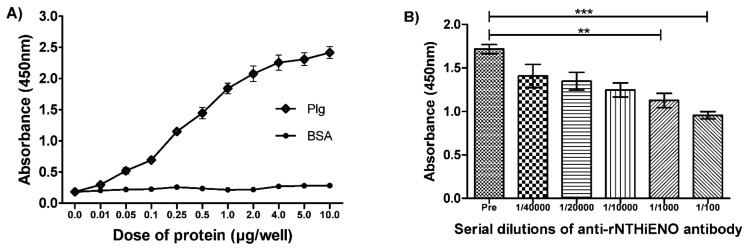
Identification of the binding of rNTHiENO to plasminogen by means of ELISA. (**A**) Plasminogen-binding assays. ELISA assays showed that the interaction between rNTHiENO and plasminogen (Plg) is concentration-dependent. Increasing amounts in concentrations (0.01–10 µg/well) of plasminogen or BSA were used as indicated in the plot. BSA was used as a negative control. Data represent the mean ± SD standard deviation of three independent experiments. (**B**) Detection by ELISA of the inhibition of the rNTHiENO–Plg interaction, using polyclonal anti-rNTHiENO antibodies at different dilutions (1/100 to 1/40,000). Pre: Preimmune serum (negative control). Data represent the mean ± SD standard deviation of three independent experiments. (*** *p* < 0.001): preimmune serum VS 1/100; (** *p* < 0.01): preimmune serum VS 1/1000.

The results observed in the far western-blot assays are consistent with the results obtained in the ELISA assays, in which rNTHiENO was significantly bound in a dose-dependent manner compared to the negative control (BSA) (Figure 6A). Furthermore, when conducting experiments to inhibit the interaction between rNTHiENO and human plasminogen by adding different dilutions of anti-rNTHiENO polyclonal antibodies (1/100–1/40,000), the results showed that the addition of increasing concentrations of anti-rNTHiENO antibody led to a dose-dependent decrease in the binding of rNTHiENO to Plg, evidencing a significant difference with the negative control (preimmune serum) (Figure 6B).

## 3. Discussion

*H. influenzae* exclusively colonizes the human nasopharynx [24]. The bacterium is classified into six capsular serotypes (a–f) and non-typeable (NT) strains. *H. influenzae* is a major pathogenic bacterium causing invasive disease [25] and remains a common cause of illness in children worldwide [26]. 

In developed countries, the annual incidence of meningitis caused by bacteria is approximately 5–10 cases per population of 100,000 [3]. 

The principal pathogen is capsulated *H. influenzae* type b (Hib). This bacterium causes many severe infections, including sepsis, epiglottitis, pneumonia, and meningitis [27]. Occasionally, capsulated strains of *H. influenzae* non-type b, mostly type a, can produce invasive infections; for instance, Hib infections [3].

In contrast, NTHi bacteria are one of the major causes of respiratory tract infections, including acute otitis media (AOM), cystic fibrosis, and community-acquired pneumonia among children, especially in developing countries. The bacteria are also known to cause chronic bronchitis and chronic obstructive pulmonary disease (COPD) in the adult lower respiratory tract [28]. Other serotypes are uncommon pathogens and are thought to have low virulence; nevertheless, some have hypothesized that with the virtual elimination of *H. influenzae* type b, other serotypes might acquire virulence traits and emerge as important pathogens [29].

In multiple studies, it has been reported that the enolase of many pathogens can facilitate their invasion and dissemination in hosts through the binding and activation of plasminogen and its subsequent conversion to active plasmin [15,30,31].

Previous studies have shown that *H. influenzae* enolase is associated with the outer membrane of the bacteria and that the enolases of typeable and non-typeable strains have a 99.54% identity. This indicates that the change between the amino acid sequence is not significant. Moreover, a putative motif for plasminogen binding (_252_FYNKENGMY_260_) was identified and found to be present in the enolases of all *H. influenzae* strains [20]. In the alignment analysis, it was observed that this motif is similar to an internal motif responsible for plasminogen binding. This motif has been well-characterized in the enolase from *S. pneumoniae* (_248_FYDKERKVY_256_) [15], and other organisms, such as *L. mexicana* [17] and *B. henselae* [6], with percentages of homology of 53.8%, 46.15%, and 38.36%, respectively. In this study, molecular interactions of NTHi enolase with host plasminogen, simulated by PatchDock, showed that both proteins present complementary forms, the motif (_252_FYNKENGMY_260_) being found in the interface region. Similar results were observed in the case of *B. henselae* and *Trichinella spiralis* [6,12]. The structure of NTHiENO was subjected to quality evaluation by means of the PROCHECK Ramachandran plot analysis, which shows that the main-chain conformations for 99.7% of amino acid residues are within the most favored or allowed regions. The normalized QMEAN value for the model was of -0.10. Z-scores of around 0.0 reflect a native-like structure and, as a rule of thumb, a QMEAN z-score below -4.0 indicates a model with low quality [32]; therefore, our model was within the typical standard deviation value. Additionally, the ProSA predicted value of the z-score -8.47 is within the range of scores typically found for native proteins, these results supporting the quality of the model.

On the other hand, the lower ACE value of -101.54, the high binding affinity (kcal/mol) of -21.3, and the low value of Kd (M) of 1.0 × 10^-15^ are results that show a good model of interaction predicted by Patchdok, and these results confirm the quality of the predicted model. Therefore, we hypothesize that both structures, enolase as well as Plg, present this complementary form and that pbmHiENO is present in the interface region, suggesting that this region of NTHiENO could be one of the main sites of binding with respect to human plasminogen.

The N-terminal region of plasminogen contains five kringle domains of 80 amino acids (K1–K5), which contain lysine binding sites [33]. Interaction with lysine leads to a conformational change, which makes it more susceptible to cleavage by plasminogen activators (Pas) [34] and so pass to its active form of plasmin.

Almost all kringle domains bind to lysine or lysine-like ligands. Interactions of the host’s Plg or its isolated kringle domains with lysine residues occur in K1, 2, 4, and 5; K1 and K4 exhibit the strongest ligand affinities, while K2 possesses the weakest affinity [35,36,37,38]. In *T. spiralis* enolase (TsENO), four lysine residues were identified, which play an important role in PLG binding, and the quadruple mutant of TsENO (Lys90Ala + Lys289Ala + Lys291Ala + Lys300Ala), in which the key lysine residues were substituted with alanine (Ala) residues, exhibited a reduction in PLG binding of nearly 50% (45.37%) [12]. 

On the other hand, one of the principal binding sites to plasminogen kringle domains on enolases studied in most of the pathogenic organisms is the C-terminal lysine [17], as was observed in the case of *S. pyogenes*, *S. pneumoniae*, [33] and *Mycobacterium tuberculosis* [39]. However, while in the case of *S. pyogenes* the C-terminal lysines are important for Plg binding, for pneumococcal enolase, Plg binding mainly depends on the internal Plg-binding site BS2 (residues 248–256), and the C-terminal lysine residue(s) is either not or only marginally involved [16,40]. *H. influenzae* enolase lacks C-terminal lysines [20]; however, it does have the internal Plg-binding site (_252_FYNKENGMY_260_). Thus, we decided to focus on this motif and submit it to blind docking using AutoDock Vina [41] in order to identify which amino acids are key to binding kringle plasminogen domains.

According to molecular docking results, the best poses with the lowest binding energies and hydrogen bonding showed that pbmHiENO (_1_EFYNKENGMYE_11_) has an affinity to K2, K3, and K5 plasminogen domains, having a binding affinity kcal/mol of -3.9, -4.4, and -4.8, respectively. We identified three key residues for plasminogen binding: one of the key amino acid residues was enoTYR_253_, which binds to plgGLU_1_ by forming hydrogen bonds. Although GLU_1_ is not found within K2, other intermolecular forces are exerted by multiple residues of plasminogen located in the K2 domain. TYR_253_ is also involved in hydrogen bonding, but in this case, with plgGLY_310_, located in K3 of Plg. The other two key residues binding to Plg were enoGLU_251_ and enoLYS_255_, binding to plgLYS_468_ and plgARG_471_, respectively, both located in the K5 domain. In addition, other residues of plasminogen located in these domains (Table 2) that participate in the interaction were also identified. On the other hand, the docking test of positive controls showed that the difference in the spatial location of the atoms of the docked pose with respect to the crystallized ligand presented an RMSD <2.0 Å. Therefore, AutoDock Vina successfully predicted the coordinates of the crystallized ligands. These results provide information of the reliability of the software; hence, it can be assumed that the results obtained with blind docking are reliable.

As already mentioned above, the role of an internal Plg-binding motif in enolases (_248_FYDKERKVY_256_) was proposed for many organisms, including parasites, yeast, and bacteria. For species such as *S. pneumoniae*, *L. mexicana*, Bifidobacterial and Candida, the role of this internal enolase motif was experimentally supported by a competitive inhibition assay with synthetic peptides and/or also by site-directed mutagenesis of the binding motif [15,17,42,43]. In the case of *S. pneumoniae*, it has been shown that LYS_251_, GLU_252_, and LYS_254_ are essential for the interaction with plasminogen. The replacement of three amino acid residues in the selected fragment of the bacterial enolase (Lys251Leu, Glu252Gly, and Lys254Leu substitutions) reduced plasminogen binding to 44% of the wild-type level [15]. These results have also been seen in Bifidobacterial enolase. By site-direct mutagenesis of LYS_251_, GLU_252_, and LYS_255_ within the BS2 homologue of *B. lactis*, BI07 enolase impaired its Plg-binding activity, suggesting that the positively charged residues Lys_251_, Lys_255_, and the negatively charged Glu_252_ are vital for Plg interaction [40]. These charges are preserved in pbmHiENO, equivalent to enoGLU_251_, enoTYR_253_ and enoLYS_255_. GLU_251_ is outside the classic characteristic plasminogen-binding motif (_252_FYNKENGMY_260_); however, it was observed that it can interact with the K5 domain. Although TYR_253_ participates in the interaction with two kringle domains (K2 and K3), we consider it important that LYS_255_ has a high probability of binding with the K5 domain due to its strategic position being exposed in the NTHiENO structure. Similar results were reported in a molecular docking of *G. intestinalis* GiENO, where LYS_266_ was shown to preferentially bind to the K5 domain of Plg [44]. With these antecedents, and the results obtained in this work, we propose that enoGLU_251_, enoTYR_253_, and enoLYS_255_ constitute the main plasminogen binding sites; however, there could be amino acids outside of pbmHiENO participating in the interaction with plasminogen or maintaining a protein folding that is favorable to their interaction with plasminogen, but this hypothesis will have to be confirmed in further studies. Meanwhile, we propose that *H. influenzae* enolase can bind to K2 and K3 but mainly K5 domains of Plg through pbmHiENO, due to the formation of two hydrogen bonds, and also because it presented the best binding affinity (kcal/mol -4.8). 

The ability of rNTHiENO to bind Plg was examined using far-western blot and ELISA assays, and the results clearly show that rNTHiENO is a Plg-binding protein in a concentration-dependent manner. This result has been reported with respect to multiple enolases in different models, such as *S. iniae* [8], *S. pyogenes* [45], *M. tuberculosis* [39], *Trichomonas vaginalis* [46], *L. mexicana* [17], and *B. henselae* [6]. 

In other studies, the interaction between plasminogen and enolase has been suggested to play a key role in the degradation of extracellular matrix proteins, in which the microbial enolase is captured by human plasminogen, and its subsequent conversion to plasmin serves as a mechanism to increase their virulence, favoring host tissue invasion [47]. Even without activation, the recruitment of plasminogen to the bacterial surface has been reported as a pivotal pathogenicity mechanism, promoting bacterial attachment to cell surfaces. A study using mice infected by the intranasal route demonstrated that plasminogen recruitment to pneumococci significantly contributes to their virulence in mice. In other studies, surface-exposed enolase was identified as a mediator for plasminogen-dependent bacterial attachment [15,48,49].

Taking into consideration previously reported studies and the results obtained from molecular docking, as well as in vitro assays in this work, it is proposed that NTHiENO–plasminogen interaction could be one of the mechanisms used by *H. influenzae* to infect and invade host cell—a mechanism for *H. influenzae* that has not been described until now. Moreover, the inhibition of rNTHiENO–Plg interactions using polyclonal anti-rNTHiENO antibodies prove to be specific. Comparison with preimmune serum (free of anti-rNTHiENO antibodies) was used to prove that the interaction of both proteins was not affected. Therefore, it is proposed that *H. influenzae* enolase could be an important therapeutic target for the development of a vaccine.

## 4. Materials and Methods

### 4.1. Enolase Sequences and Alignments

The amino acid sequence of NTHiENO (GenBank: MF405339.1) and the enolase sequences of several microorganisms in which it has already been shown that the enzyme has an internal motif involved in plasminogen (Plg)-binding were used. These microorganisms were: *Streptococcus pneumoniae* (GenBank: VDG78305.1), *Leishmania mexicana* (GenBank: ABA64522.1), and *Bartonella henselae* (GenBank: ATP12173.1). The sequences were aligned using multiple sequence alignment with CLUSTAL O (1.2.4) (https://www.ebi.ac.uk/Tools/msa/clustalo/, accessed on 1 October 2021).

### 4.2. Analyses of Protein–Protein Docking

The three-dimensional model of NTHiENO homology was obtained by Phyre2 [21]. Once the model was obtained, quality and stereochemistry were evaluated using the PROCHECK program [50]. The QMEAN (Qualitative Model Energy Analysis) program of the SWISS-MODEL was used for the estimation of the best reliable model quality [32], and the ProSA (Protein Structure Assessment) tool was used to check the overall quality of the model [51]. 

The crystallized structure of human plasminogen was obtained from the Protein Data Bank (ID 4A5T). Molecular docking of protein–protein interactions between NTHiENO and human plasminogen was analyzed using PatchDock [52,53]. The first 10 best coupling results were considered, based on the best score and the lowest atomic contact energy (ACE). The selection of the best model, based on the calculation of the lowest ACE value, presented the putative plasminogen-bind motif in the interface region. The binding mode was visualized by Chimera [22]. Moreover, the select model was submitted to analysis to evaluate the binding affinity (ΔG) and dissociation constant (Kd) by the PRODIGY server [54]. 

### 4.3. Obtaining the Internal Binding Motif to Plg (pbmHiENO)

From the three-dimensional model of NTHiENO described above, the putative internal binding motif to Plg, _251_EFYNKENGMYE_261_ (pbmHiENO), was cut with the GaussView 5.0 program [55], adding an amino acid in both ends. To neutralize the charges of amino acids from the ends that were cut off, the caps CT3 (N-methyl) and ACE (acetyl) to the N- and C-terminal regions were added, respectively, by VMD software [56]. This peptide was used as a ligand to perform the blind docking assays. 

### 4.4. Blind Docking pbmHiENO–Plg

Both the plasminogen molecule (protein) and pbmHiENO (ligand) were prepared first with the Chimera program [22] and later with AutoDock tools. The blind docking was executed in the AutoDock Vina program [41], using as a target each region of the five kringle domains (K1–K5) of the human plasminogen. The search for conserved domains was carried out through the NCBI conserved domain search platform (https://www.ncbi.nlm.nih.gov/Structure/cdd/wrpsb.cgi, accessed on 1 October 2021). Several couplings were performed, and the best models were selected based on better binding affinity (kcal/mol) and hydrogen bond formation.

### 4.5. Docking Positive Controls

To validate the reliability of the AutoDock Vina program [41] in relation to the prediction of interactions, selected co-crystallized molecules with experimental ligands were selected. Plasmin (the active form of plasminogen) and two other molecules with similar characteristics to plasminogen in terms of biological function and to the chemical nature of the ligand (pbmHiENO) were used. All molecules were prepared using Chimera [22]. The selected molecules that served as positive controls were plasmin EC 3.4.21.7 (Protein Data Bank: ID 5UGG) with its ligand (Protein Data Bank: ID 89M) [57], alpha-thrombin EC 3.4.21.5 (Protein Data Bank: ID 1HDT) with its ligand (Protein Data Bank: ID 0E7) [58], and blood coagulation factor Xa EC 3.4. 21.5 (Protein Data Bank: ID 1LPG) with its ligand (Protein Data Bank: ID IMA) [59]. Better binding affinity kcal/mol was evaluated, and, finally, the root mean square deviation (RMSD) of the docked pose was obtained with respect to the original ligand, calculated using SPDV software [60].

### 4.6. Plasminogen-Binding Assay by Far-Western Blot

To detect enolase–plasminogen interactions, far-western blot assays were performed as described in Hall [61]. Briefly, the human plasminogen (Sigma-Aldrich, Saint Louis, MO, USA) (5 and 10 μg) and rNTHiENO (3 μg), obtained according to [20], were migrated in an SDS-PAGE (10%) and later electrotransferred to a nitrocellulose membrane (Bio-Rad, Inc, Hercules, CA, USA). The membrane was blocked with blocking buffer (2% non-fat powdered milk, 0.1% Tween-20 in PBS) by 1 h/RT, after which it was incubated with 10 μg/mL of rNTHiENO or 10 μg/mL of BSA as a negative control in blocking buffer, overnight at 4 °C, followed by three washes with blocking buffer. rNTHiENO binding was detected by incubating the membrane with polyclonal anti-rNTHiENO antibodies as the first antibody type and with goat anti-mouse IgG alkaline phosphatase-conjugated (Novex^®^ by Life Technologies, Van Allen Way Carlsbad, CA, USA) as the secondary antibody. The bound antibodies were revealed using NBT (nitro blue tetrazolium) and BCIP (5-bromo-4-chloro-3-indolyl-phosphate) (Thermo Fisher Scientific, Waltham, MA, USA). 

### 4.7. Detection of Interactions of rNTHiENO with Plasminogen by ELISA

Briefly, a 96-well plate was coated with 1.0 μg/well of rNTHiENO diluted in 100 μL of PBS (phosphate-buffered saline) and incubated overnight at 4 °C. After that, three washes with PBS-Tween (PBST) 0.5% were performed, and the wells were blocked with 2% BSA for 2 h at 37 °C. The plate was washed three times again and incubated with 0.01–10 μg/well of human plasminogen (Sigma-Aldrich, USA) diluted in 100 μL of PBS or with BSA as a negative control at the same concentrations for 2 h at 37 °C. Binding protein was detected with polyclonal anti-Plg antibodies as first antibody and goat anti-mouse IgG-HRP-conjugated as the secondary antibody. 

Inhibition experiments were performed by adding polyclonal anti-rNTHiENO antibodies (serial dilutions from 1/100 to 1/40,000) prior to the addition of Plg (1.0 μg/well). These antibodies were obtained in a previous work [20]. The interaction was visualized using 3,3’,5,5’-Tetramethylbenzidine (TMB; Sigma-Aldrich), and the reaction was stopped with the addition of 50 μL of 0.5 M sulfuric acid, and, finally, the OD was read at 450 nm. All experiments were repeated three times.

### 4.8. Generation of Anti-Plg Polyclonal Antibodies

For the immunization scheme, four mice BALB/C, were bled at the tail to collect preimmune serum; later, they were immunized with 20 μg of human plasminogen (Sigma-Aldrich, USA) with TiterMax^®^ Gold Adjuvant (Sigma-Aldrich) by intramuscular route, followed by three boosters with the same concentrations, a period of eight days between each one. At the end of the immunization scheme, the mice were bled to obtain the hyperimmune serum. The polyclonal antibodies were tested by western blot, where they showed recognition by purified human plasminogen. The experimental procedure (Protocol Code: 100522577-UALVIEP-18/1, 30 October 2018) was approved by the Claude Bernal Animal Care and Use Committee of the Benemerita Universidad Autonoma de Puebla. The mice were housed in a controlled environment and managed according to the National Institutes of Health Guide to the Care and Use of Experimental Animals and following the guidelines of the Norma Oficial Mexicana: Guide for the Care and Use of Laboratory Animals (NOM-062-ZOO-1999). 

## 5. Conclusions

The results obtained in this work revealed that recombinant enolase of *H. influenzae* is capable of binding human plasminogen. Moreover, three residues identified in the predicted plasminogen-binding domain in NTHiENO showed a high probability of interaction with the K2, K3, and K5 domains of Plg. The importance of demonstrating the interaction between both proteins, in addition to describing what could be the key amino acids that carry out the interaction between both proteins, are important results that allow us to propose enolase as one mechanism that could be used by *H. influenzae* to infect and invade host cells. Therefore, the enolase of *H. influenzae* could be considered as a virulence factor that allows this bacterium to increase its repertoire of pathogenicity mechanisms. 

In summary, to our knowledge, this is the first work that describes the *H. influenzae* enolase–human plasminogen interaction by computational methods and experimental assays. 

## Figures and Tables

**Table 1:** Blind docking results for NTHiENO residues binding to kringle domains of plasminogen.

Target Kringle Plg Domain	Residue in NTHiENO	Residue in pbmHiENO	Residue Plg	Hydrogen Bonding Interactions	Binding Energy (kcal/mol)
K2	TYR_253_	TYR_3_	GLU_1_	1	-3.9
K3	TYR_253_	TYR_3_	GLY_310_	1	-4.4
K5	LYS_255_ GLU_251_	LYS_5_ GLU_1_	ARG_471_ LYS_468_	2	-4.8

**Table 2:** Plasminogen amino acids participating in the pbmHiENO–Plg interaction.

Kringle Domain	Plasminogen Amino Acid Residue
K2	GLU_1_, ILE_178_, SER_179_, LYS_180_, ASP_239_, and PRO_241_
K3	VAL_272_, GLY_310_, LYS_311_, ARG_324_, TRP_325_, GLU_326_, TYR_327_, TYR_713_, GLU_714_, PHE_715_, LEU_730_, and ASN_769_
K5	LYS_468_, Gly_469_, ARG_471_, LYS_473_, PRO_503_, ARG_504_, ALA_505_, GLY_506_, GLU_508_, and LYS_509_

**Table 3:** Summary results of docking positive controls.

Receptor (ID PDB)	Crystalized Ligand (ID PDB)	Binding Energy (kcal/mol)	RMSD Crystallized Ligand/Docked Pose
Plasmin (5UGG)	89M	-7.8	0.61 Å
Alpha-thrombin (1HDT)	0E7	-7.2	0.65 Å
Factor Xa (1LPG)	IMA	-9.1	0.68 Å

## Data Availability

The datasets used and/or analyzed during the current study are available from the corresponding author upon reasonable request.
